# Cerebroprotein hydrolysate‐I protects senescence‐induced by D‐galactose in PC12 cells and mice

**DOI:** 10.1002/fsn3.2333

**Published:** 2021-05-17

**Authors:** Lin Zhu, Yingjuan Liu, Xiaolin Wu, Yuqian Ren, Qinghua Zhang, Leiming Ren, Yunliang Guo

**Affiliations:** ^1^ Institute of Cerebrovascular Diseases Taishan Scholars Construction Project Excellent Innovative Team of Shandong Province Medical Research Center The Affiliated Hospital of Qingdao University Qingdao China; ^2^ Department of Neurology Shandong Second Provincial General Hospital Jinan China; ^3^ Institute of Chinese Integrative Medicine Hebei Medical University Shijiazhuang China

**Keywords:** cerebroprotein hydrolysate‐I, D‐galactose, mice, PC12 cells, senescence, telomerase

## Abstract

Cerebroprotein hydrolysate‐I (CH‐I)，a mixture of peptides extracted from porcine brain tissue，has shown a neuroprotective effect, but its role in brain senescence is unclear. In the present study, we established a senescence model of PC12 cells and mice to investigate the effect of CH‐I on brain senescence via JAK2/STAT3 pathway. The results showed that CH‐I could improve cell viability, inhibit the apoptosis of cells, and reduce the senescence‐positive cells induced by D‐galactose. In *vivo*, CH‐I improved the learning ability and memory of aging mice, reduced neuronal damage in mice hippocampus. Mechanism studies showed that CH‐I could adjust BDNF protein expressions, activate JAK2/STAT3 pathway, and finally enhance telomerase activity. All these findings indicated that CH‐I showed a neuroprotective effect against brain senescence. These results might provide further reference and support for the application of CH‐I in delaying aging.

## Graphical abstract

Cerebroprotein hydrolysate‐I prevent D‐gal induced senescence of PC12 cells Cerebroprotein hydrolysate‐I prevent hippocampal senescence in mice induced by D‐gal Cerebroprotein hydrolysate‐I regulate telomerase by JAK2/STAT3 pathway to delay senescence

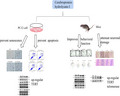

## 1 INTRODUCTION

The aging population has become a worldwide concern. Evidence suggests that brain aging can increase the possibility of developing neurodegenerative disease (Coutu et al., 2017). Given the incidence of neurodegenerative disease trends to rise year by year (Arribas et al., 2018), researchers have been trying to seek safe and effective treatments based on the mechanisms of aging. Although the pathogenesis of aging is highly complex, the previous study has shown that telomere attrition is an important factor in accelerating aging (López‐Otín et al., 2013), and the maintenance of telomere length is mainly depended on telomerase activity. Telomeres attrition and decreasing of telomerase activity in brain may cause aging and death of neurons, leading to cognitive decline (Qi et al., 2019). The deletion of telomerase affected brain function in mice, which showed neuron loss with memory impairment (Rolyan et al., 2011) and anxious behavior (Lee et al., 2010). Telomerase reactivation in adult mice could improve age‐related decline in cognitive performance (Jaskelioff et al., 2011), indicating that telomerase activation may be an effective method to delay brain aging.

Telomerase is composed of telomerase reverse transcriptase (TERT) and telomerase RNA component (TERC). TERT is regarded as the main regulatory subunit of telomerase, and up‐regulation or phosphorylation of TERT can activate telomerase activity. TERT undergoes transcriptional activation by a lot of signals, among which signal transducer and activator of transcription 3 (STAT3) are considered as a prominent activator (Kumar et al., 2018; Wang et al., 2012). Although STAT3 and phosphorylated STAT3 activate telomerase in tumor cells, it still can play a positive role in aging and repair nerve injury (Benito et al., 2017; Park et al., 2013). The activation of STAT3 depends on the JAK2 (Janus kinase2) /STAT3 signaling pathway, which is considered as a signaling cascade that has a prominent role in immune function and cancer development. Increasing evidence also suggests that JAK2/STAT3 signaling pathway plays an important role in the brain, especially in combination with neurotrophic factors. Studies have shown that brain‐derived neurotrophic factor (BDNF) can promote nerve regeneration and improve nerve injury by activating the JAK/STAT pathway (Benito et al., 2017). Activation and phosphorylation of STAT3 by increasing BDNF can also improve memory deficits in rats (Alawdi et al., 2017). The activation of JAK2/STAT3 may be useful in treating cognitive impairment associated with aging‐related disorders (Park et al., 2013). Therefore, the JAK2/STAT3 pathway may play a role in regulating brain senescence and telomerase activity.

Cerebroprotein hydrolysate‐I (CH‐I) is a mixture of peptides extracted from porcine brain tissue which has been shown an effect in inhibiting neuroinflammation and free radical formation and can promote neurogenesis (Guan et al., 2019). In addition, it can pass through the blood‐brain barrier to improve neuronal survivals and repair neurons (Rockenstein et al., 2015). Therefore, CH‐I is widely regarded as a potential neurotrophic and neuroprotective drug in the treatment of vascular dementia, stroke and AD (Cui et al., 2019; Li et al., 2013). Based on the above, we speculate that CH‐I may act on telomerase through JAK2/STAT3 pathway to delay aging. However, the effects of CH‐I on brain senescence have not been yet reported. In this study, we try to investigate the neuroprotective effects and possible mechanisms of CH‐I against brain senescence.

## 2 MATERIALS AND METHODS

### 2.1 Cell culture

PC12 cells were purchased from iCell Institute of Biotechnology (Shanghai, China) and maintained in RPMI 1,640 medium (HyClone, USA) with 10% heat‐inactivated horse serum (Solarbio, China), 5% fetal bovine serum (Gibco, USA), and 1% penicillin‐streptomycin (Solarbio, China) at 37°C in a humidified atmosphere with 5% CO_2_.

### 2.2 D‐gal‐induced cell injury

The cells were seeded into 96‐well plates and incubated for 24 hr, and then treated with different concentrations (5, 10, 15, 20, 25, and 30 mg/ml, for 24 hr, 48 hr, and 72 hr) of D‐gal (purity ≥99%, Aladdin, China). Cell viability was measured by Cell counting kit‐8 (CCK‐8 assay, Solarbio, China). 10 μl of CCK‐8 solution was added to each well and incubated for 2.5 hr at 37°C. Absorbance was measured at 450 nm with a Microplate Reader (SYNERGYH1, Bio‐Tek Instruments, Winooski, VT, USA). The suitable inhibitory concentration and time of D‐gal were selected based on the result.

### 2.3 Cell viability assay

PC12 cells were seeded into 96‐well plates and incubated for 24 hr. Different concentrations (15, 30, 60, 120, and 240 μg/ml) of CH‐I (0190501‐1, 30 mg/ampoule, Hebei Zhitong, China, a Chinese FDA ratification code of GuoYaoZhunZi‐H20051737/H20051738) were added to the cells for 12 hr, 24 hr, and 48 hr. Then, cells were treated with 20 mg/ml of D‐gal for 48 hr except for the control group. Cell viability was evaluated by the CCK‐8 assay as described above.

### 2.4 Senescence associated β‐galactosidase staining

Cellular senescence was detected by senescence‐associated β‐galactosidase (SA‐β‐gal) staining kit (Beyotime, China). PC12 cells were plated in 6‐well plates. After grouped and treated as described above, the cells were stained with β‐galactosidase staining solution and incubated overnight at 37°C (free CO_2_). The number of positive cells was calculated under a light microscope (IX‐70, Olympus, Tokyo, Japan).

### 2.5 Cell apoptosis assay

Cell apoptosis was determined by Annexin V‐EGFP/PI cell apoptosis detection kit (TransGen, China). PC12 cells which treated as indicated were harvested and resuspended in 100 μl of binding buffer. Then cells were incubated with 5 μl Annexin V‐FITC and 5 μl PI for 15 min at room temperature protected from light. The samples were analyzed by Flow Cytometry (CytoFLEX, Beckman Coulter).

### 2.6 Animals and treatment

Forty‐eight male C57/BL6N mice (2‐month old) were purchased from Weitong Lihua Experimental Animal Technology (Beijing, China) (SCXK: 2016‐0006). The animal experiments were approved by the Ethics Committee of Qingdao University Medical College and The National Institutes of Health Guide for the Care and Use of Laboratory Animals was used as a guide for the design of all animal‐related studies (QYFY WZLL 2020‐10‐26). All animals were maintained in a controlled environment with constant temperature (20 ± 3) °C and relative humidity (60 ± 10)%. The mice had free access to food and water. The mice were randomly divided into four groups (*n* = 12), including the control group, model group, CH‐I groups (3 and 6 mg/kg). The mice in control group were given an intraperitoneal injected of normal saline, while others were given D‐gal (150 mg/kg) once a day for 8 weeks. As described previously (Nam et al., 2019), after 3 weeks of D‐gal treatment, CH‐I was injected separately to the mice once daily for 5 weeks.

### 2.7 Morris water maze

The Morris Water Maze (MWM) test was performed to measure spatial learning and memory of mice (Ceglia et al., 2015). Briefly, the mice were given a four‐quadrant hidden platform test per day for 5 consecutive days and a probe trial on day 6. For the hidden platform test, the mice were given 60 s to reach the hidden platform and allowed to stay on the platform for 30 s. The platform was removed on day 6 for the probe trial, and each mouse was allowed to explore for 60 s. The swimming trajectory and the time spent in the target platform quadrant were recorded and analyzed by the video tracking analysis system.

### 2.8 Open field experiment test

Following MWM test for 7 days, the open field experiment was used to observe the spontaneous exploration activity of mice after the administration of drugs (Zheng et al., 2019). Each mouse was placed in one of the four corner squares facing the wall. Then, their behavior was recorded on video for 5 min. The instrument recorded the latency time for the mouse to walk out of the central and surrounding areas. Each mouse was wiped off after completing the experiment to avoid leaving odors and dirt that could interfere with the results of the experiment.

### 2.9 HE staining

Four mice were randomly selected from each group. Each mouse was perfused through the heart to harvest the brain. Immersion‐fixed brain (hippocampus) tissues were routinely embedded in paraffin and sectioned at 7 μm. The hippocampal sections were dewaxed and rehydrated, then were stained by a hematoxylin‐eosin (HE) staining kit (Solarbio, China) according to the manufacturer's instruction. Finally, all the staining sections were used an inverted microscope (Olympus, Tokyo, Japan) to obtain the images. The degree of damage is denoted by the denatured cell index (DCI = number of denatured cells/total number of cells).

### 2.10 Western blot analysis

Total proteins from cells and hippocampal tissues of mice were extracted and the protein content was determined. Samples were separated by dodecyl sulfate‐polyacrylamide gel electrophoresis (SDS‐PAGE) and electrotransferred to polyvinylidene fluoride (PVDF) membranes (Millipore, Burlington, MA, USA). The membranes were blocked with 5% skim milk at room temperature for 2 hr and incubated at 4°C overnight with the primary antibodies (BDNF 1:1,000, Zen, China) (JAK2 1:1,000, STAT3 1:1,000, p‐STAT3 1:1,000, β‐actin 1:10,000, Affinity, USA) (p‐JAK2 1:1,000, Boster, China) (TERT 1:500, Novus, USA). Then, the membranes were washed and incubated with the horseradish peroxidase‐secondary antibody (goat anti‐rabbit IgG 1:10,000, goat anti‐mouse IgG 1:10,000) at room temperature for 1.5 hr. Protein signals were detected using enhanced chemiluminescence reagents (Millipore, Burlington, MA, USA). Images were acquired by a BioSpectrum version 810 imaging system (UVP) and analyzed using the Quantity One software.

### 2.11 Telomerase activity assay

Telomerase activity was measured by Telomerase PCR‐ELISA kit (Roche, Germany) according to the manufacturer's protocols. Briefly, extracts from the hippocampal tissues of C57 mice were obtained using lysate. Then, the extracts were added to reaction mixture and transferred to a thermal cycler (Bio‐Rad, CA, USA). Measurement protocol times were as follows: 25°C for 30 min, 94°C for 5 min, 30 cycles at 94°C for 30 s, 50°C for 30 s, and 72°C for 90 s. Cell extracts that were previously heat‐inactivated (at 95°C for 10 min) were used as a negative control, while protein extracts supplied with the kit served as a positive control. Upon dilution of PCR product with the Hybridization buffer, the mixtures were added onto 96‐well plates. Following denaturation and hybridization at 37°C for 2 hr, anti‐digoxigenin peroxidase conjugate and TMB substrate were used for the ELISA assay, and the absorbance at 450 nm was measured using a microplate reader (Bio‐Tek Instruments, Winooski, VT, USA). Samples with relative absorbance values more of than 0.2 were considered positive.

### 2.12 Statistical analysis

All statistical analyses were analyzed by GraphPad Prism version 8. One‐way analysis of variance was used to assess multiple groups of data and Student's *t*‐test was used to compare between two groups. *p* ≤ .05 was considered statistically significant.

## 3 RESULTS

### 3.1 CH‐I reduce D‐gal‐induced injury in PC12 cells

**FIGURE 1:**
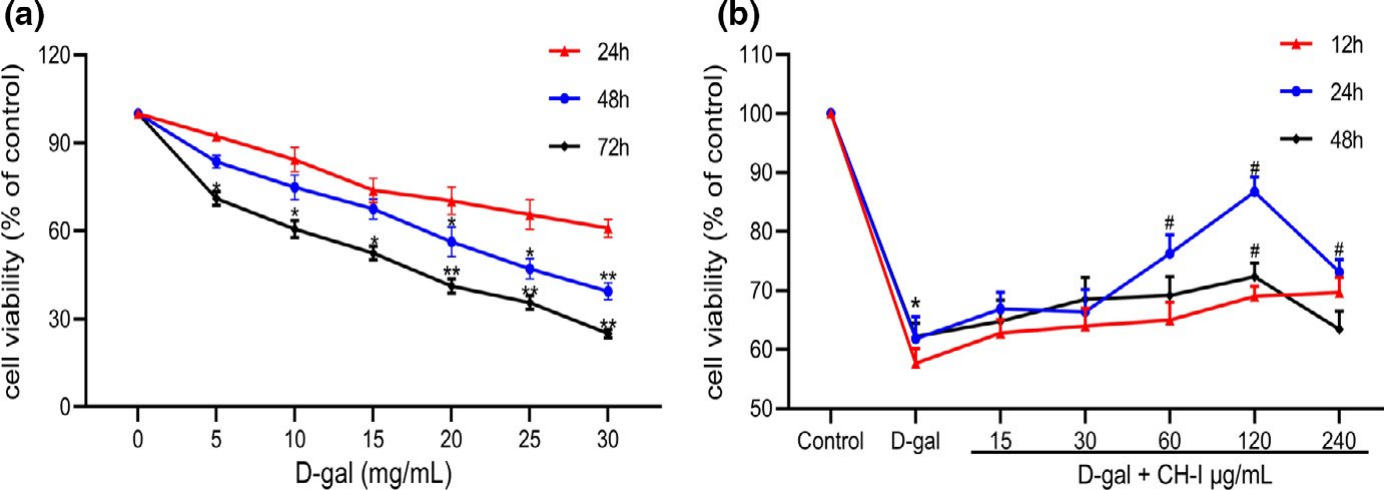
CH‐I reduce D‐gal‐induced injury in PC12 cells. (a) The inhibitory effect of the D‐gal on PC12 cell viability. PC12 cells were treated with various concentrations of D‐gal. (b) CH‐I inhibited the damage of PC12 cells induced by D‐galactose. *p < .05 and **p < .01 vs control group; ^#^ *p* < .05 vs D‐gal group

D‐gal in the range of 20–30 mg/ml can significantly decreased cell viability (*p* < .05), which was decreased to (56.27 ± 2.92) % by 20 mg/ml of D‐gal for 48 hr (Figure 1a). Therefore, D‐gal at 20 mg/ml for 48 hr was selected. The test was given to explore the protective effect of CH‐I (15, 30, 60, 120, and 240 μg/ml for 12 hr, 24 hr, and 48 hr). CH‐I could prevent cells from the damage of D‐gal as shown by improved cell viability, while treatment with 60 and 120 μg/ml CH‐I showed a significantly protective effect compared with D‐gal group (*p* < .05) (Figure 1b). Therefore, the two concentrations of CH‐I were selected for further experiments.

### 3.2 CH‐I prevents senescence‐induced by D‐gal in PC12 cells

**FIGURE 2:**
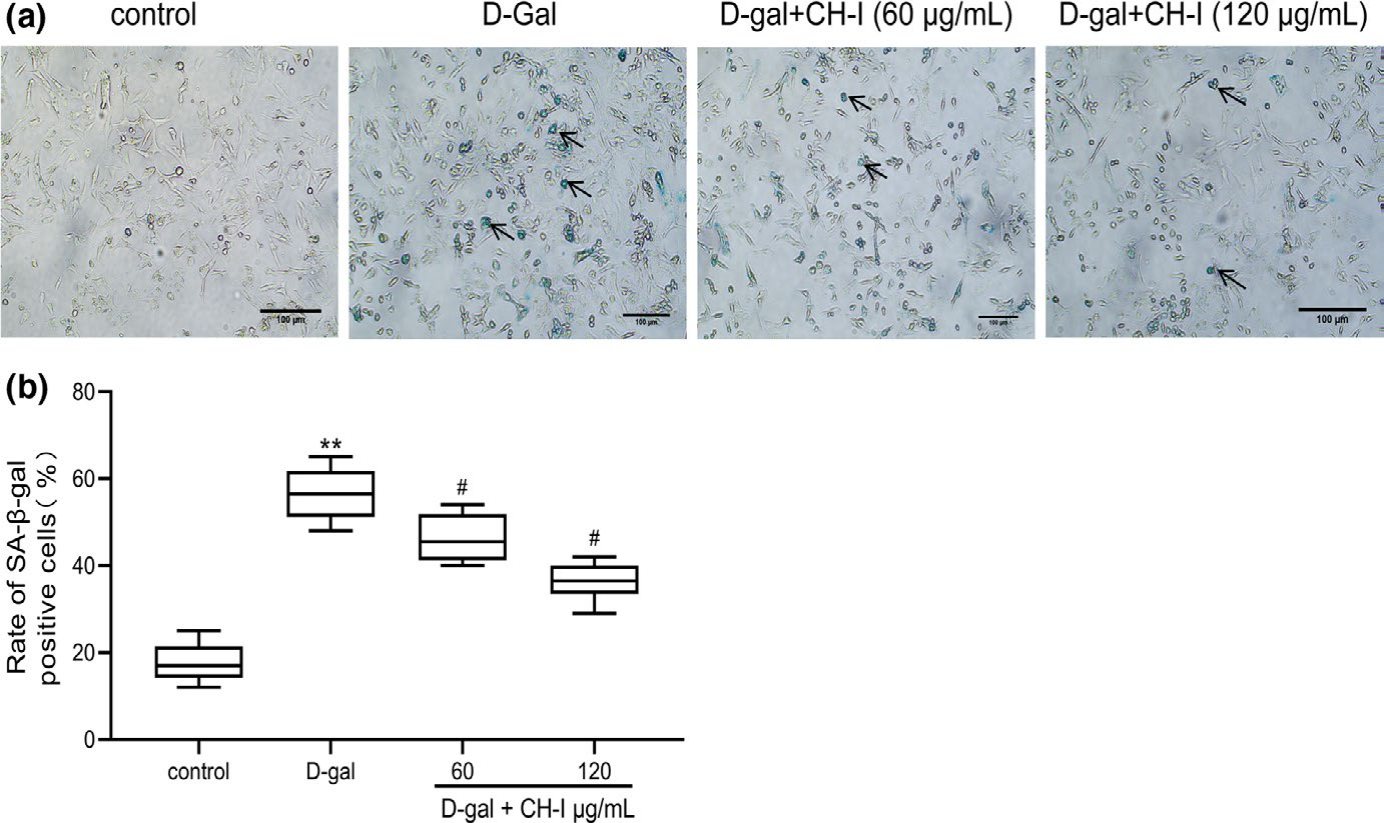
CH‐I prevents senescence induced by D‐gal in PC12 cells. (a) Photomicrographs showed SA‐β‐Galpositive cells (blue staining) in PC12 cells (magnification, ×100). (b) The rate of SA‐β‐Gal positive cells were calculated per group. **p < .001 vs control group; ^#^ *p* < .05 vs D‐gal group

The SA‐β‐gal staining results show that the numbers of blue senescent cells in D‐gal group were obviously increased, which was significantly reduced by CH‐I (*p* < .01). Compared with D‐gal group, the proportion of SA‐β‐Gal‐positive cells was markedly decreased in treatment with CH‐I (*p* < .05) (Figure 2).

### 3.3 CH‐I prevents apoptosis induced by D‐Gal in PC12 cells

**FIGURE 3:**
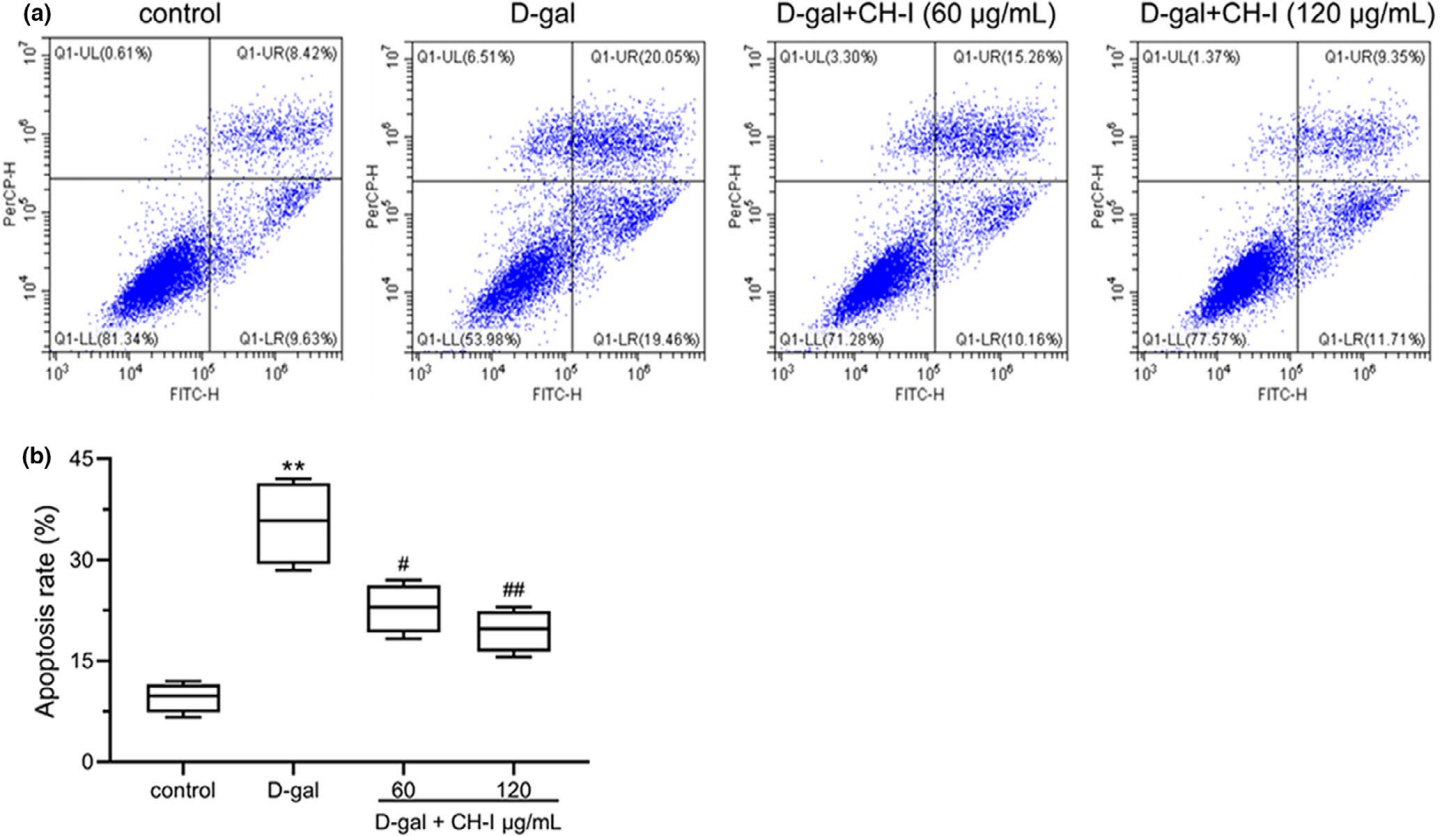
CH‐I prevents apoptosis induced by D‐gal in PC12 cells. (a) Annexin V/PI staining and flow cytometry analysis of cell apoptosis. (b) The average value of the percentages of apoptotic cells from repeated. **p < .01 vs control group; ^#^ *p* < .05 and ^##^ *p* < .01 vs D‐gal group

Flow cytometry was used to detect the effect of CH‐I on apoptosis in PC12 cells. The result showed that CH‐I increased the apoptosis rate of PC12 cells damaged by D‐gal compared to the control group (*p* < .01). And compared with D‐gal group, the apoptosis rate was significantly reduced by the CH‐I (*p* < .05) (Figure 3).

### 3.4 CH‐I promotes TERT expression and activates JAK2/STAT3 signaling in PC12 cells

**FIGURE 4:**
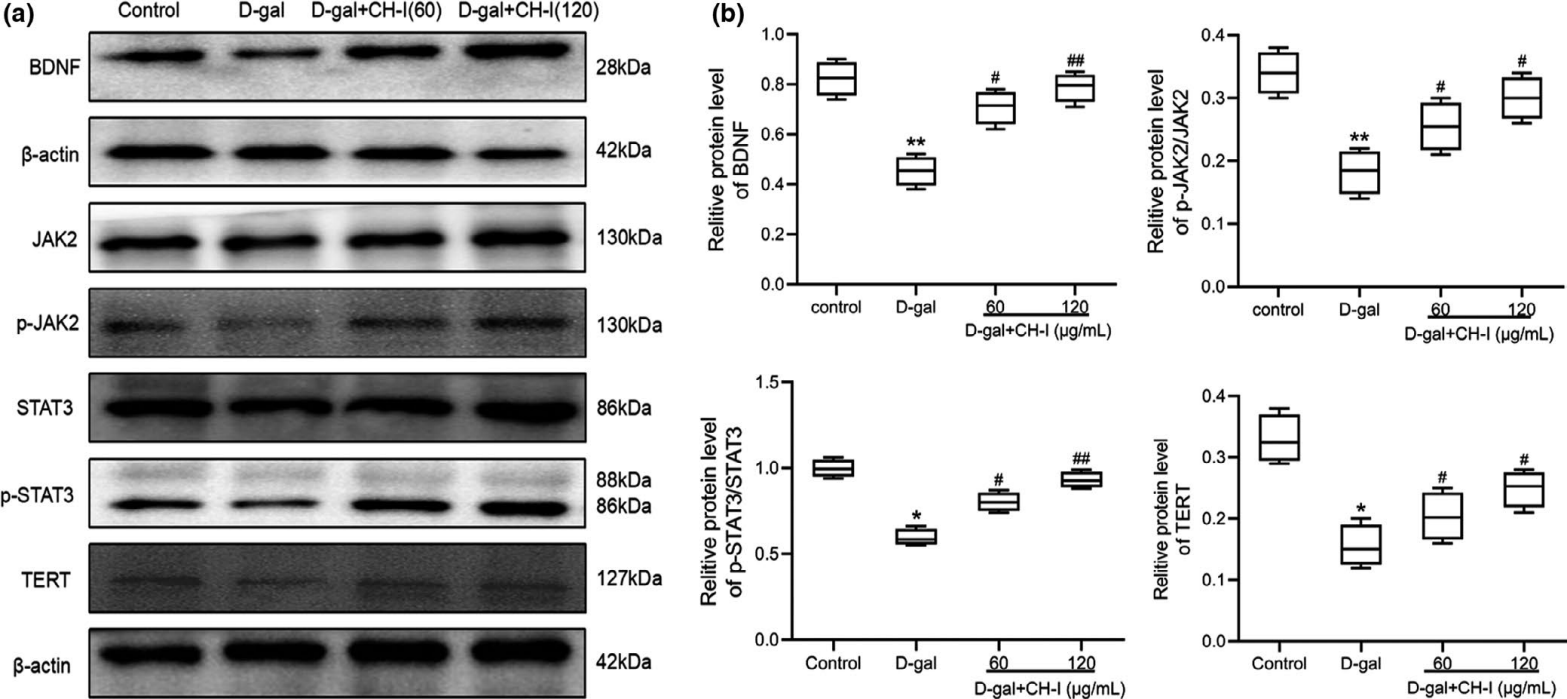
Effects of CH‐I on related protein expressions of PC12 cells induced by D‐gal. (a) The original bands of BDNF, JAK2, p‐JAK2, STAT3, p‐STAT2, TERT and β‐actin. β‐actin was considered as loading controls. (b) The ratio of different proteins to β‐actin was calculated by the band density. *p < .05, **p < .01 vs control group; ^#^ *p* < .05 and ^##^ *p* < .01 vs D‐gal group

To verify whether JAK2/STAT3 pathway was involved in protective effects of CH‐I, we further analyze protein characterization of PC12 cells using Western blotting. The results showed that compared with control group, the expression of BDNF was decreased, and the phosphorylation levels of JAK2 and STAT3 were down‐regulated after D‐gal induction. The expression of TERT protein was also reduced (*p* < .05). But after treatment with CH‐I, the expression of BDNF was significantly increased, and the levels of p‐JAK2, p‐Stat3, and TERT were raised (*p* < .05) (Figure 4). Meanwhile, the total protein levels of JAK2 and STAT3 were relatively constant in all the groups.

### 3.5 CH‐I improved the Cognition and Exploratory Dysfunction of Aging Mice

**FIGURE 5:**
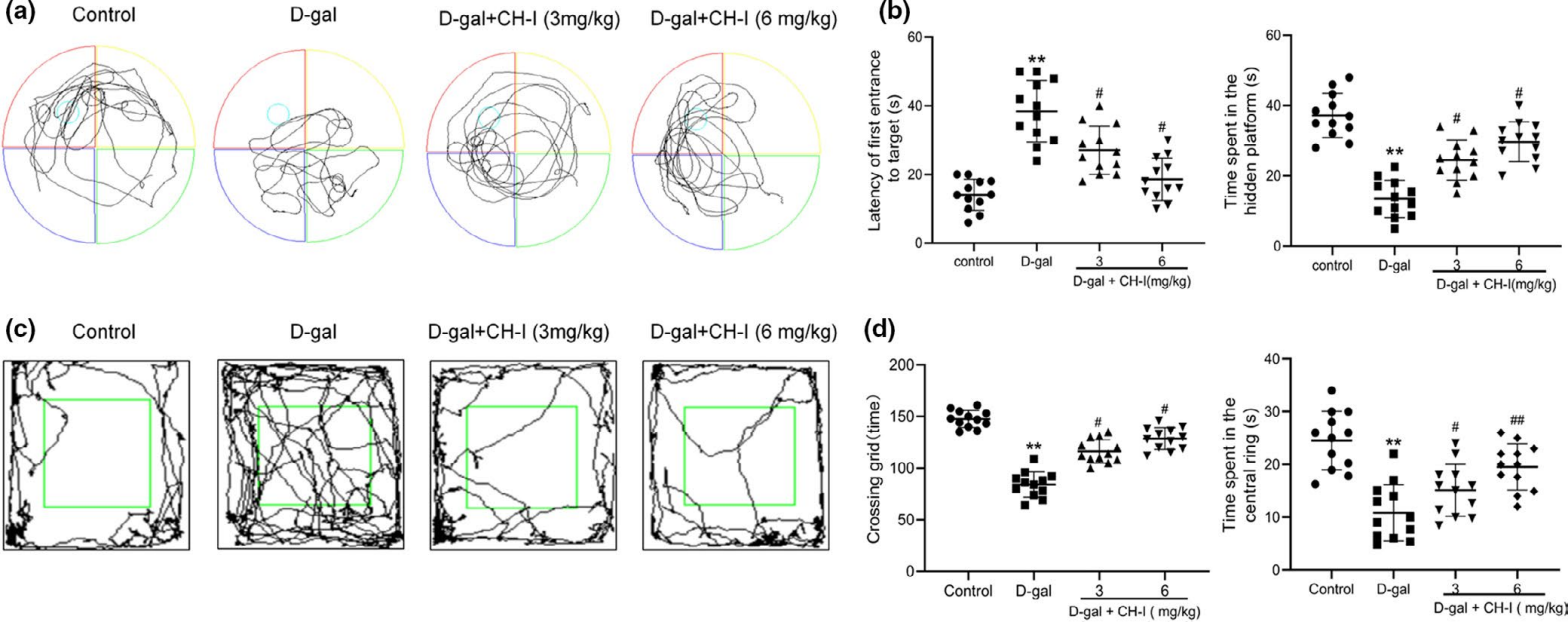
CH‐I improved the cognition and exploratory dysfunction of aging mice. (a) Effects of CH‐I on terms of search strategy in the MWM test. (b) The time spent in the hidden platform and the latency of first entrance to target of aging mice in the probe trial. (c) Mice exploring behavior in the open field test. (d) The time spent in center of the open filed box. **p < .01 vs control group; ^#^ *p* < .05 and ^##^ *p* < .01 vs D‐gal group

In terms of search strategy, the search strategy of model group was mainly random and aimless, while the data in CH‐I groups showed a straight line and purpose especially at 6mg/kg, suggesting the embodiment of strong spatial learning and memory ability (Figure 5a). In the probe trial (Figure 5b), compared with control group, the latency of first entrance to target and the time spent in the target platform quadrant in model group were significantly reduced (*p* < .01), which was reversed by CH‐I (*p* < .05). The above results indicated that CH‐I significantly improved the cognition deficit of aging mice.

The open‐field experiment was used to assess autonomic activities and exploratory behaviors. A high frequency of these behaviors indicates increased locomotion and exploration (Figure 5c). The results showed (Figure 5d) that D‐gal‐treated mice exhibited a significant reduction in open field activity compared with control group of mice (*p* < .01). Compared with D‐gal group, CH‐I groups significantly improved autonomic activities (*p* < .05). The results indicated that CH‐I could ameliorate the behavioral impairments caused by D‐gal.

### 3.6 CH‐I attenuated neuronal damage in the hippocampus of aging mice

**FIGURE 6:**
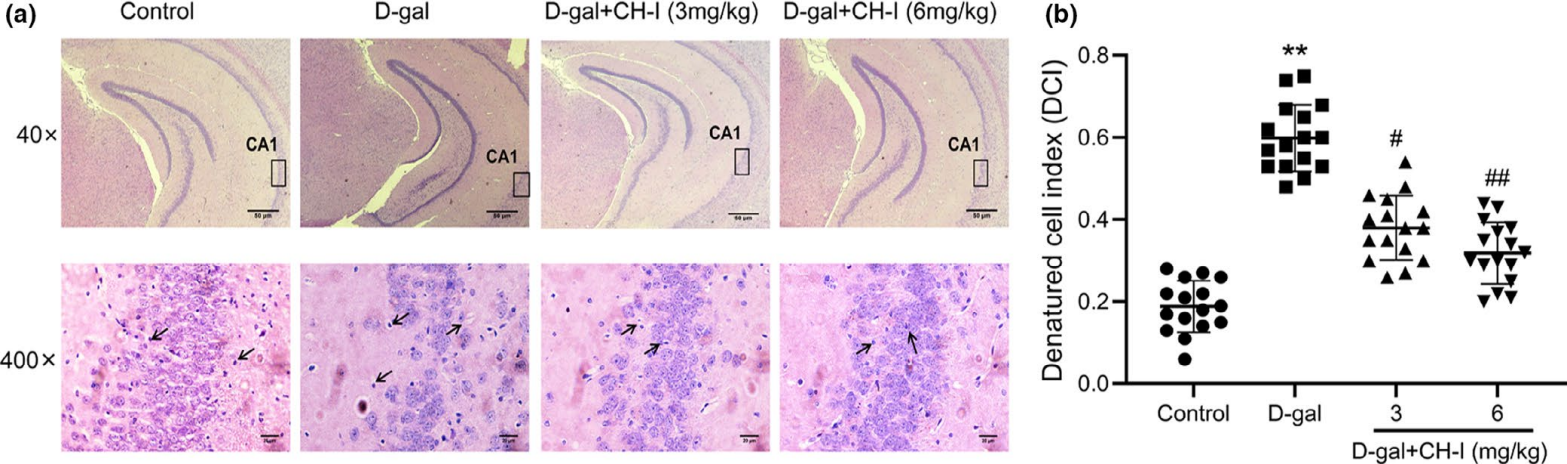
CH‐I attenuated neuronal damage in the hippocampus of aging mice. (a) Representative images of HE staining of the hippocampus (40× and 400× magnification). (b) The denatured cell index of hippocampal region. **p < .01 vs control group; ^#^ *p* < .05, ^##^ *p* < .01 vs D‐gal group

HE staining was performed to evaluate the neuroprotective effect of CH‐I treatment. As shown in Figure 6a, the neurons in hippocampus were neatly arranged in control group, while the mice in D‐gal group showed that the nuclei of hippocampal neurons were pyknotic and hyperchromatic and the array of neurons were disordered and incompact with part of them formed cavities. The DCI of D‐gal group was significantly higher than that in the control group (*p* < .01) (Figure 6b). These results suggested that D‐gal could cause pathological changes of hippocampal neurons in mice. The hippocampal morphologies of CH‐I groups were restored. Compared with D‐gal group, the neurons in CH‐I groups tended to be arranged in a more orderly manner, and the DCI was significantly reduced (*p* < .05), suggesting the effect of CH‐I on reducing the neuron damages of the aging brain.

### 3.7 CH‐I increased the expression of TERT and telomerase activity in hippocampus of aging mice

**FIGURE 7:**
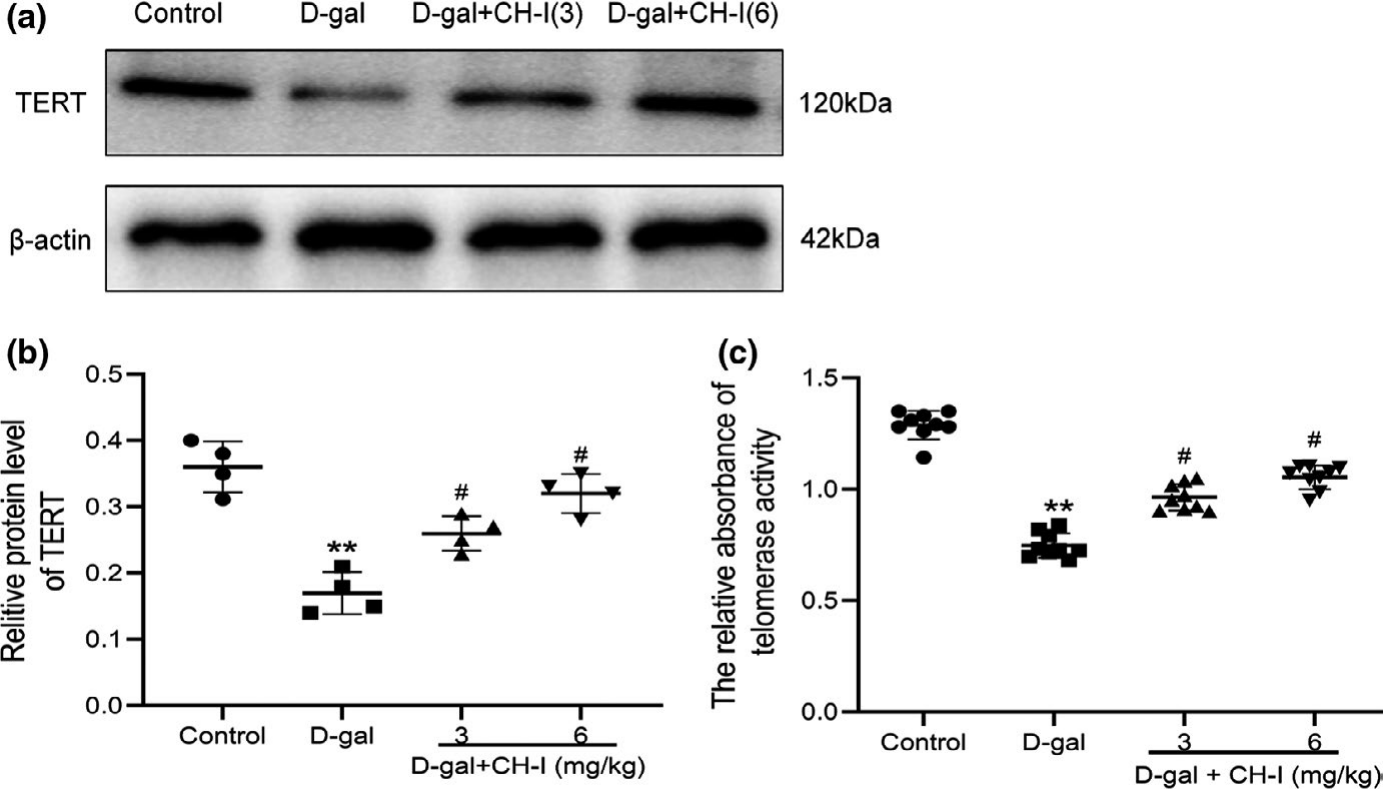
CH‐I promoted TERT expression and telomerase activity. (a) The original bands of TERT and β‐actin. β‐actin was considered as loading controls. (b) The effect of CH‐I on TERT protein expressions in hippocampus of aging mice. (c) The relative absorbance of telomerase activity was measured by PCR‐ELISA. **p < .01 vs control group; ^#^ *p* < .05 vs D‐gal group

We evaluated the effect of CH‐I on TERT protein expression and telomerase activities in the hippocampus of aging mice. Firstly, we detected the protein expression of TERT. The protein expression levels were declined in hippocampus in D‐gal group (Figure 7a, 7b). Then, we examined telomerase activity using a PCR‐ELISA assay. The results showed that compared with control group, the telomerase activity was significantly decreased after D‐gal treatment (*p* < .01). However, the telomerase activity in hippocampus of CH‐I treated mice was significantly increased compared with that of the D‐gal group (*p* < .05).

### 3.8 CH‐I promoted the activation of JAK2/STAT3 signaling in D‐gal‐induced aging mice

**FIGURE 8:**
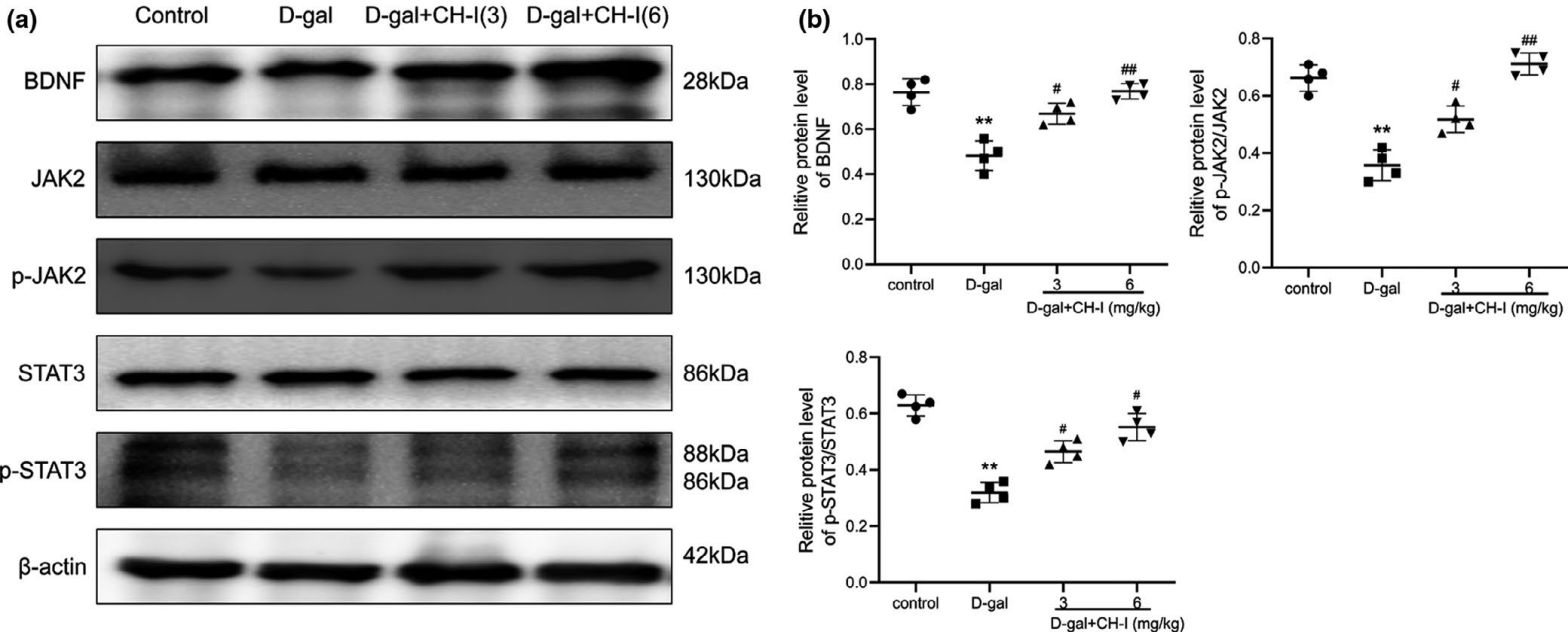
Effects of CH‐I on JAK2/STAT3 signaling pathway related proteins expressions of hippocampus in D‐gal‐induced aging mice. (a) The original bands of BDNF, JAK2, p‐JAK2, STAT3, p‐STAT2, and β‐actin. β‐actin was considered as loading controls. (b) The ratio of different proteins to β‐actin was calculated by the band density. **p < .01 vs control group; ^#^ *p* < .05, ^##^ *p* < .01 vs D‐gal group

We then assessed the expression of JAK2/STAT3 signaling pathway‐related proteins in hippocampus of aging mice after CH‐I treatment. Similar results to the cell level，the expression of BDNF in the hippocampus was significantly increased (*p* < .05), and the decline of p‐JAK2, p‐Stat3 proteins caused by D‐gal could be reversed (*p* < .05) (Figure 8a, 8b).

## 4 DISCUSSION

A large number of studies have shown that abnormal accumulation of D‐gal in cells and mice can lead to nervous system senescence (Hsieh et al., 2011; Kaviani et al., 2017). Therefore, PC12 cell damage and C57/BL6N mice senescence‐induced by D‐gal were selected as models to investigate the prevention and treatment effect of CH‐I on senility. The results showed that the positive rate of cell aging and apoptosis of PC12 cells in model group was significantly increased, the ability of learning and memory and autonomous activity of mice in model group were significantly decreased, and neuronal damage in the hippocampus was significantly aggravated.

CH‐I is a mixture containing bioactive peptides derived from the porcine brain, which has similar effects to neurotrophic factors (such as brain‐derived neurotrophic factor, nerve growth factor), and has been considered as a potential neuroprotective drug for the treatment of age‐related nervous system diseases (Tatebayashi et al., 2003; Xing et al.,^2014^). Our study demonstrated for the first time that CH‐I could significantly improve cell survival at concentrations of 60 and 120 μg/ml, alleviated D‐gal‐induced PC12 cell senescence and apoptosis. D‐gal‐induced aging mice were accompanied by behavioral disorders such as the decrease of learning and memory and autonomic activities, while hippocampus was obviously damaged. Our study found that in animal models, CH‐I (3 and 6 mg/kg) could improve the ability of learning and memory, increase the autonomic activity of aging mice, and significantly reduce hippocampal neuronal damage caused by D‐gal in mice. These results suggest that CH‐I may play a role in the aging of the nervous system.

Accumulated evidence support that telomerase reactivation is an effective way to delay aging (Cheung et al., 2014; Shay, 2016; Yu et al., 2014; Cheng et al., 2019). The role of telomerase in brain aging has been confirmed, and the inactivation of telomerase is associated with many neurodegenerative diseases (Eitan et al., 2012; Shoeb et al., 2020), making telomerase a potential therapeutic target for age‐related neurological diseases. Telomerase reverse transcriptase (TERT), as the key structure and main regulatory subunit of telomerase, plays an important role in the regulation of telomerase activity. We found that TERT protein expression was significantly decreased in D‐Gal‐induced PC12 cells and mice hippocampal tissues, and a significant increase in TERT expression was also observed following treatment with CH‐I.

Increased telomerase expression may have some beneficial effects on the development and progression of neurodegenerative diseases. In rodent brains, telomerase activity becomes undetectable by postnatal day 10, the TERT mRNA maintains at a lower level until adulthood (Klapper et al., 2001). Other studies confirmed the telomerase activity in the adult hippocampus, cerebellum and cortex (Lee et al., 2010; Mamdani et al., 2015). Compared with control group, telomerase activity in hippocampus of D‐gal‐induced aging mice was significantly decreased. After CH‐I treatment, telomerase activity in the hippocampus was significantly increased compared with the model mice. Meanwhile, the learning and memory ability of the aging mice were dramatically improved. Therefore, we considered that CH‐I can antagonize the cognitive dysfunction associated with aging induced by D‐gal in mice, which may be related to the enhancement of telomerase activity.

TERT can be up‐regulated by a variety of transcription factors. In this study, we focused on exploring the ways to regulate TERT expression and telomerase activity. STAT3 is a transcription factor that plays a key role in cytokine and growth factor signaling, it has been shown that as a transcription factor of TERT, STAT3 can up‐regulate TERT expression (Guo et al., 2012; Wang et al., 2012) and plays a role in aging and repair nerve injury (Benito et al., 2017; Park et al., 2013). STAT3 activation is dependent on the JAK2/STAT3 signaling pathway, and the activation of JAK2/STAT3 may be useful in treating cognitive impairment associated with aging‐related disorders (Park et al., 2013). Based on this, we think that the JAK2/STAT3 pathway may play a role in regulating aging and telomerase activity. Previous studies have shown that neurotrophic factors such as BDNF can activate the JAK2/STAT3 pathway (Qi et al., 2019). Therefore, we detected the expression of BDNF, p‐JAK2 and p‐STAT3 proteins in PC12 cells and hippocampal tissues of aging mice after the addition of CH‐I. In this study, we found that BDNF levels were significantly increased after treatment with CH‐I, which is consistent with previous research (Alvarez et al., 2016). And CH‐I reversed the decrease of p‐JAK2, p‐STAT3 induced by D‐gal, which was consistent with TERT protein expression and telomerase. The results suggested that CH‐I showed anti‐aging and neuroprotective effects, which may be related to the enhancement of BDNF expression, activation of JAK/STAT pathway, and ultimately activation of telomerase activity. This study is expected to provide new approaches for delaying senescence of the nervous system and preventing age‐related diseases.

## 5 CONCLUSION

In conclusion, D‐gal induced senescence and apoptosis of PC12 cells and resulted in a hippocampal tissue senescence damage in mice. Our results suggest that CH‐I can improve the senescence of PC12 cells and mice hippocampal tissue, which may be achieved by increasing telomerase activity.

## CONFLICT OF INTEREST

The authors declare no conflict of interest.

## AUTHOR CONTRIBUTION

**Lin Zhu:** Conceptualization (equal); Data curation (lead); Formal analysis (lead); Investigation (equal); Methodology (equal); Resources (equal); Software (equal); Validation (equal); Visualization (lead); Writing‐original draft (lead); Writing‐review & editing (lead). **Yingjuan Liu:** Conceptualization (equal); Data curation (supporting); Formal analysis (supporting); Investigation (equal); Methodology (equal); Resources (supporting); Software (supporting); Visualization (supporting); Writing‐original draft (equal); Writing‐review & editing (supporting). **Xiaolin Wu:** Conceptualization (supporting); Data curation (supporting); Investigation (supporting); Methodology (supporting); Resources (equal); Software (supporting); Writing‐original draft (supporting). **Yuqian Ren:** Conceptualization (supporting); Data curation (equal); Formal analysis (supporting); Investigation (supporting); Methodology (supporting); Resources (equal); Visualization (supporting); Writing‐original draft (supporting). **Qinghua Zhang:** Conceptualization (supporting); Formal analysis (supporting); Methodology (supporting); Software (supporting); Writing‐original draft (supporting). **Leiming Ren:** Conceptualization (supporting); Formal analysis (supporting); Funding acquisition (supporting); Investigation (supporting); Methodology (equal); Software (equal); Writing‐original draft (supporting). **yunliang Guo:** Conceptualization (lead); Formal analysis (lead); Funding acquisition (lead); Investigation (lead); Methodology (lead); Project administration (lead); Resources (lead); Software (lead); Supervision (lead); Validation (lead); Writing‐original draft (lead); Writing‐review & editing (lead).

6

## References

[fsn32333-bib-0001] Alawdi, S. H. , El‐Denshary, E. S. , Safar, M. M. , Eidi, H. , David, M. O. , & Abdel‐Wahhab, M. A. (2017). Neuroprotective effect of nanodiamond in Alzheimer's disease rat model: A pivotal role for modulating NF‐κB and STAT3 signaling. Molecular Neurobiology, 54(3), 1906–1918. 10.1007/s12035-016-9762-0 26897372

[fsn32333-bib-0002] Alvarez, X. A. , Alvarez, I. , Iglesias, O. , Crespo, I. , Figueroa, J. , Aleixandre, M. , Linares, C. , Granizo, E. , Garcia‐Fantini, M. , Marey, J. , Masliah, E. , Winter, S. , Muresanu, D. , & Moessler, H. (2016). Synergistic increase of serum BDNF in Alzheimer patients treated with cerebrolysin and donepezil: Association with cognitive improvement in ApoE4 cases. International Journal of Neuropsychopharmacology, 19(6), pyw024. 10.1093/ijnp/pyw024 PMC4926802 27207906

[fsn32333-bib-0003] Arribas, R. L. , Romero, A. , Egea, J. , & de los Ríos, C. (2018). Modulation of serine/threonine phosphatases by melatonin: Therapeutic approaches in neurodegenerative diseases. British Journal of Pharmacology, 175(16), 3220–3229. 10.1111/bph.14365 29781146 PMC6057903

[fsn32333-bib-0004] Benito, C. , Davis, C. M. , Gomez‐Sanchez, J. A. , Turmaine, M. , Meijer, D. , Poli, V. , Mirsky, R. , & Jessen, K. R. (2017). STAT3 controls the long‐term survival and phenotype of repair Schwann cells during nerve regeneration. Journal of Neuroscience, 37(16), 4255–4269. 10.1523/JNEUROSCI.3481-16.2017 28320842 PMC5413174

[fsn32333-bib-0005] Ceglia, I. , Reitz, C. , Gresack, J. , Jung‐Hyuck, A. , Bustos, V. , Bleck, M. , Zhang, X. Z. , Martin, G. , Simon, S. M. , Nairn, A. C. , Greengard, P. , & Kim, Y. (2015). APP intracellular domain‐WAVE1 pathway reduces amyloid‐β production. Nature Medicine, 21(9), 1054–1059. 10.1038/nm.3924 PMC4560977 26280122

[fsn32333-bib-0006] Cheng, L. , Yuan, B. , Ying, S. , Niu, C. , Mai, H. , Guan, X. , Yang, X. , Teng, Y. , Lin, J. , Huang, J. , Jin, R. , Wu, J. , Liu, B. , Chang, S. , Wang, E. , Zhang, C. , Hou, N. , Cheng, X. , Xu, D. , … Ye, Q. (2019). PES1 is a critical component of telomerase assembly and regulates cellular senescence. Science. Advances, 5(5), eaav1090. 10.1126/sciadv.aav1090 PMC6520020 31106266

[fsn32333-bib-0007] Cheung, H. H. , Liu, X. , Canterel‐Thouennon, L. , Li, L. , Edmonson, C. , & Rennert, O. M. (2014). Telomerase protects werner syndrome lineage‐specific stem cells from premature aging. Stem Cell Reports, 2(4), 534–546. 10.1016/j.stemcr.2014.02.006 24749076 PMC3986587

[fsn32333-bib-0008] Coutu, J. P. , Lindemer, E. R. , Konukoglu, E. , & Salat, D. H. (2017). Two distinct classes of degenerative change are independently linked to clinical progression in mild cognitive impairment. Neurobiology of Aging, 54(1), 1–9. 10.1016/j.neurobiolaging.2017.02.005 28286328 PMC5519085

[fsn32333-bib-0009] Cui, S. , Chen, N. , Yang, M. , Guo, J. , Zhou, M. , Zhu, C. , & He, L. (2019). Cerebrolysin for vascular dementia. Cochrane Database of Systematic Reviews, 2019(11), CD008900. 10.1002/14651858.CD008900.pub3 PMC6844361 31710397

[fsn32333-bib-0010] Eitan, E. , Tichon, A. , Gazit, A. , Gitler, D. , Slavin, S. , & Priel, E. (2012). Novel telomerase‐increasing compound in mouse brain delays the onset of amyotrophic lateral sclerosis. EMBO Molecular Medicine, 4(4), 313–329. 10.1002/emmm.201200212 22351600 PMC3376858

[fsn32333-bib-0011] Guan, X. , Wang, Y. , Kai, G. , Zhao, S. , Huang, T. , Li, Y. , Xu, Y. , Zhang, L. , & Pang, T. (2019). Cerebrolysin ameliorates focal cerebral ischemia injury through neuroinflammatory inhibition via CREB/PGC‐1α Pathway. Frontiers in Pharmacology, 10, 1245. 10.3389/fphar.2019.01245 31695614 PMC6818051

[fsn32333-bib-0012] Guo, N. , Cheng, D. , Li, Z. H. , Zhou, Q. B. , Zhou, J. J. , Lin, Q. , Zeng, B. , Liao, Q. , & Chen, R. F. (2012). Transfection of HCVc improves hTERT expression through STAT3 pathway by epigenetic regulation in Huh7 cells. Journal of Cell Biochemistry, 113(11), 3419–3426. 10.1002/jcb.24218 22688977

[fsn32333-bib-0013] Hsieh, H. M. , Wu, W. M. , & Hu, M. L. (2011). Genistein attenuates D‐galactose‐induced oxidative damage through decreased reactive oxygen species and NF‐κB binding activity in neuronal PC12 cells. Life Sciences, 88(1–2), 82–88. 10.1016/j.lfs.2010.10.021 21056584

[fsn32333-bib-0014] Jaskelioff, M. , Muller, F. L. , Paik, J. H. , Thomas, E. , Jiang, S. , Adams, A. , Sahin, E. , Kost‐Alimova, M. , Protopopov, A. , Cadiñanos, J. , Horner, J. W. , Maratos‐Flier, E. , & Depinho, R. A. (2011). Telomerase reactivation reverses tissue degeneration in aged telomerase‐deficient mice. Nature, 469(7328), 102–106. 10.1038/nature09603 21113150 PMC3057569

[fsn32333-bib-0015] Kaviani, E. , Rahmani, M. , Kaeidi, A. , Shamsizadeh, A. , Allahtavakoli, M. , Mozafari, N. , & Fatemi, I. (2017). Protective effect of atorvastatin on D‐galactose‐induced aging model in mice. Behavioural Brain Research, 34(1), 55–60. 10.1016/j.bbr.2017.07.029 28750834

[fsn32333-bib-0016] Klapper, W. , Shin, T. , & Mattson, M. P. (2001). Differential regulation of telomerase activity and TERT expression during brain development in mice. Journal of Neuroscience Research, 64(3), 252–260. 10.1002/jnr.1073 11319769

[fsn32333-bib-0017] Kumar, A. , Tripathy, M. K. , Pasquereau, S. , Al Moussawi, F. , Abbas, W. , Coquard, L. , Khan, K. A. , Russo, L. , Algros, M. P. , Valmary‐Degano, S. , Adotevi, O. , Morot‐Bizot, S. , & Herbein, G. (2018). The human cytomegalovirus strain DB activates oncogenic pathways in mammary epithelial cells. EBioMedicine, 30(2018), 167–183. 10.1016/j.ebiom.2018.03.015 29628341 PMC5952350

[fsn32333-bib-0018] Lee, J. , Jo, Y. S. , Sung, Y. H. , Hwang, I. K. , Kim, H. , Kim, S. Y. , Yi, S. S. , Choi, J. S. , Sun, W. , Seong, J. K. , & Lee, H. W. (2010). Telomerase deficiency affects normal brain functions in mice. Neurochemical Research, 35(2), 211–218. 10.1007/s11064-009-0044-3 19685288

[fsn32333-bib-0019] López‐Otín, C. , Blasco, M. A. , Partridge, L. , Serrano, M. , & Kroemer, G. (2013). The hallmarks of aging. Cell, 153(6), 1194–1217. 10.1016/j.cell.2013.05.039 23746838 PMC3836174

[fsn32333-bib-0020] Mamdani, F. , Rollins, B. , Morgan, L. , Myers, R. M. , Barchas, J. D. , Schatzberg, A. F. , Watson, S. J. , Akil, H. , Potkin, S. G. , Bunney, W. E. , Vawter, M. P. , & Sequeira, P. A. (2015). Variable telomere length across post‐mortem human brain regions and specific reduction in the hippocampus of major depressive disorder. Translational Psychiatry, 5(9), e636. 10.1038/tp.2015.134 26371764 PMC5068804

[fsn32333-bib-0021] Nam, S. M. , Seo, M. , Seo, J. S. , Rhim, H. , Nahm, S. S. , Cho, I. H. , Chang, B. J. , Kim, H. J. , Choi, S. H. , & Nah, S. Y. (2019). Ascorbic acid mitigates D‐galactose‐induced brain aging by increasing hippocampal neurogenesis and improving memory function. Nutrients, 11(1), 176. 10.3390/nu11010176 PMC6356429 30650605

[fsn32333-bib-0022] Park, S. J. , Shin, E. J. , Min, S. S. , An, J. , Li, Z. , Hee Chung, Y. , Hoon Jeong, J. , Bach, J. H. , Nah, S. Y. , Kim, W. K. , Jang, C. G. , Kim, Y. S. , Nabeshima, Y. , Nabeshima, T. , & Kim, H. C. (2013). Inactivation of JAK2/STAT3 signaling axis and downregulation of M1 mAChR cause cognitive impairment in klotho mutant mice, a genetic model of aging. Neuropsychopharmacol, 38(8), 1426–1437. 10.1038/npp.2013.39 PMC3682136 23389690

[fsn32333-bib-0023] Qi, Y. , Li, R. , Xu, L. , Yin, L. , Xu, Y. W. , Han, X. , & Peng, J. Y. (2019). Neuroprotective effect of dioscin on the aging arain. Molecules, 24(7), 1247. 10.3390/molecules24071247 PMC6479446 30935017

[fsn32333-bib-0024] Rockenstein, E. , Desplats, P. , Ubhi, K. , Mante, M. , Florio, J. , Adame, A. , Winter, S. , Brandstaetter, H. , Meier, D. , & Masliah, E. (2015). Neuro‐peptide treatment with cerebrolysin improves the survival of neural stem cell grafts in an APP transgenic model of Alzheimer disease. Stem Cell Research, 15(1), 54–67. 10.1016/j.scr.2015.04.008 26209890

[fsn32333-bib-0025] Rolyan, H. , Scheffold, A. , Heinrich, A. , Begus‐Nahrmann, Y. , Langkopf, B. H. , Hölter, S. M. , Vogt‐Weisenhorn, D. M. , Liss, B. , Wurst, W. , Lie, D. R. , Thal, D. R. , Biber, K. , & Rudolph, K. L. (2011). Telomere shortening reduces Alzheimer's disease amyloid pathology in mice. Brain, 134(Pt 7), 2044–2056. 10.1093/brain/awr133 21672962

[fsn32333-bib-0026] Shay, J. W. (2016). Role of telomeres and telomerase in aging and cancer. Cancer Discovery, 6(6), 584–593. 10.1158/2159-8290.CD-16-0062 27029895 PMC4893918

[fsn32333-bib-0027] Shoeb, M. , Mustafa, G. M. , Kodali, V. K. , Smith, K. , Roach, K. A. , Boyce, G. , Meighan, T. , Roberts, J. R. , Erdely, A. , & Antonini, J. M. (2020). A possible relationship between telomere length and markers of neurodegeneration in rat brain after welding fume inhalation exposure. Environmental Research, 180, 108900. 10.1016/j.envres.2019.108900 31711660 PMC6899181

[fsn32333-bib-0028] Tatebayashi, Y. , Lee, M. H. , Li, L. , Iqbal, K. , & Grundke‐Iqbal, I. (2003). The dentate gyrus neurogenesis: A therapeutic target for Alzheimer's disease. Acta Neuropathologica, 105(3), 225–232. 10.1007/s00401-002-0636-3 12557008

[fsn32333-bib-0029] Wang, Y. Y. , Sun, G. , Luo, H. , Wang, X. F. , Lan, F. M. , Yue, X. , Fu, L. S. , Pu, P. Y. , Kang, C. S. , Liu, N. , & You, Y. P. (2012). MiR‐21 modulates hTERT through a STAT3‐dependent manner on glioblastoma cell growth. CNS Neuroscience & Therapeutics, 18(9), 722–728. 10.1111/j.1755-5949.2012.00349.x 22709411 PMC6493453

[fsn32333-bib-0030] Xing, S. , Zhang, J. , Dang, C. , Liu, G. , Zhang, Y. , Li, J. , Fan, Y. , Pei, Z. , & Zeng, J. (2014). Cerebrolysin reduces amyloid‐β deposits, apoptosis and autophagy in the thalamus and improves functional recovery after cortical infarction. Journal of the Neurological Sciences, 337(1–2), 104–111. 10.1016/j.jns.2013.11.028 24315581

[fsn32333-bib-0031] Yu, T. Y. , Kao, Y. W. , & Lin, J. J. (2014). Telomeric transcripts stimulate telomere recombination to suppress senescence in cells lacking telomerase. Proceedings of the National Academy of Sciences of the United States of America, 111(9), 3377–3382. 10.1073/pnas.1307415111 24550456 PMC3948247

[fsn32333-bib-0032] Zhang, L. I. , Chopp, M. , Meier, D. H. , Winter, S. , Wang, L. , Szalad, A. , Lu, M. , Wei, M. , Cui, Y. , & Zhang, Z. G. (2013). Sonic hedgehog signaling pathway mediates cerebrolysin‐improved neurological function after stroke. Stroke, 44(7), 1965–1972. 10.1161/STROKEAHA.111.000831 23696546

[fsn32333-bib-0033] Zheng, P. , Zeng, B. , Liu, M. , Chen, J. , Pan, J. , Han, Y. , Liu, Y. , Cheng, K. , Zhou, C. , Wang, H. , Zhou, X. , Gui, S. , Perry, S. W. , Wong, M. L. , Licinio, J. , Wei, H. , & Xie, P. (2019). The gut microbiome from patients with schizophrenia modulates the glutamate‐glutamine‐GABA cycle and schizophrenia‐relevant behaviors in mice. Science. Advances., 5(2), eaau8317. 10.1126/sciadv.aau8317 PMC6365110 30775438

